# Disease-specific heterogeneity of C-reactive protein across 21 hematologic disorders reflects divergent inflammatory and hematopoietic phenotypes

**DOI:** 10.3389/fimmu.2026.1941789

**Published:** 2026-09-17

**Authors:** Honghao Guo, Jiale Zhou, Xin Wang, Dandan Wang, Lingyun Sun

**Affiliations:** 1 Department of Rheumatology and Immunology, Nanjing Drum Tower Hospital, Clinical College of Nanjing Medical University, Nanjing, China; 2 Department of Hematology, Nanjing Drum Tower Hospital, Affiliated Hospital of Nanjing University Medical School, Nanjing, China; 3 Department of Rheumatology and Immunology, The Affiliated Drum Tower Hospital of Nanjing University Medical School, Nanjing, China

**Keywords:** C-reactive protein, hematologic disorders, leukemia, lymphoma, multiple myeloma, NHANES, systemic inflammation

## Abstract

**Background:**

C-reactive protein (CRP) is routinely used to quantify systemic inflammation, yet its interpretation in hematologic disorders is complicated by marked biological heterogeneity. We characterized pretreatment CRP distributions across a broad hematologic disease spectrum and examined whether CRP–laboratory associations differed among clinically relevant subtypes.

**Methods:**

This retrospective study included 4,823 patients aged ≥14 years with 21 hematologic disorders evaluated at Nanjing Drum Tower Hospital between 2009 and 2023. Baseline CRP and routine laboratory variables obtained before disease-directed therapy were analyzed across diseases and within acute myeloid versus acute lymphoblastic leukemia, chronic myeloid versus chronic lymphocytic leukemia, Hodgkin versus non-Hodgkin lymphoma, and multiple myeloma with versus without extramedullary disease. Spearman correlations and CRP-quartile analyses were used to characterize covariation with hematologic, biochemical, and nutritional measures. An unweighted exploratory analysis of six nonconsecutive National Health and Nutrition Examination Survey cycles compared participants reporting leukemia or lymphoma with non-cancer participants.

**Results:**

Baseline CRP differed substantially across the 21 disorders (global *P* < 0.001), with the highest median values in hemophagocytic lymphohistiocytosis (44.6 mg/L) and thrombotic thrombocytopenic purpura (25.4 mg/L). Among 758 patients with acute leukemia subtypes, CRP was higher in acute myeloid than acute lymphoblastic leukemia (18.5 vs 9.2 mg/L; *P* = 0.004); increasing CRP was accompanied by lower albumin and higher lactate dehydrogenase in both subtypes. CRP did not differ between chronic myeloid and chronic lymphocytic leukemia (4.7 vs 3.8 mg/L; *P* = 0.150), although its laboratory correlation profile was broader in chronic myeloid leukemia. Hodgkin lymphoma showed higher CRP than non-Hodgkin lymphoma (17.2 vs 5.4 mg/L; *P* < 0.001), together with stronger leukocyte and platelet responses across CRP quartiles. In multiple myeloma, CRP was identical in patients with and without extramedullary disease (3.8 vs 3.8 mg/L; *P* = 0.712). In the NHANES sample, hs-CRP was highest among participants reporting leukemia, intermediate among those reporting lymphoma, and lowest among non-cancer participants; within the combined hematologic cancer group, hs-CRP correlated inversely with hemoglobin and albumin.

**Conclusions:**

CRP is not a uniform marker across hematologic disorders. Its baseline concentration and accompanying laboratory phenotype depend on disease context, with prominent elevations in hyperinflammatory and selected aggressive disorders but limited value for identifying extramedullary myeloma. CRP should be interpreted as a context-dependent adjunct rather than a stand-alone diagnostic or prognostic marker.

## 1 Introduction

C-reactive protein (CRP) is a highly conserved pentraxin and one of the most widely used clinical biomarkers of acute and chronic inflammation. Hepatic CRP synthesis is principally induced by interleukin-6, with contributions from interleukin-1β and other inflammatory signals, and circulating concentrations can rise rapidly in response to infection, tissue injury, autoimmunity, and malignancy (1–3). Beyond its value as a soluble readout, the biological effects attributed to CRP are conformation dependent: native pentameric CRP participates in complement regulation and opsonization, whereas locally generated monomeric CRP has been associated with endothelial activation, cell injury, pro-inflammatory lipid signaling, and amplified tissue inflammation (2–7). These mechanistic observations should not be conflated with routine serum CRP assays, which quantify systemic concentration rather than CRP conformation or tissue localization.

Inflammation is integral to many hematologic disorders but arises through different mechanisms. Acute leukemias and aggressive lymphomas may combine tumor-cell turnover, cytokine release, marrow failure, tissue infiltration, and treatment-independent metabolic stress. Chronic leukemias and plasma-cell neoplasms may have lower systemic inflammatory activity despite a substantial clonal burden. Hyperinflammatory syndromes such as hemophagocytic lymphohistiocytosis (HLH) and thrombotic microangiopathies such as thrombotic thrombocytopenic purpura (TTP) introduce additional, non-neoplastic pathways of cytokine and endothelial activation. Consequently, the same CRP concentration may have different clinical meaning across diseases, and a single universal threshold is unlikely to capture this heterogeneity.

Most available studies have evaluated CRP in one malignancy, often in selected prognostic cohorts or in combination with albumin and other inflammatory indices (8–11). Disease-specific CRP heterogeneity has also been described across autoimmune conditions (12), but cross-disease comparisons within hematology using a uniform sampling window are uncommon. Differences within broad diagnostic categories—such as acute myeloid leukemia (AML) versus acute lymphoblastic leukemia (ALL), chronic myeloid leukemia (CML) versus chronic lymphocytic leukemia (CLL), Hodgkin lymphoma (HL) versus non-Hodgkin lymphoma (NHL), or multiple myeloma (MM) with versus without extramedullary disease (EMD)—remain incompletely characterized. Correlations between CRP and routine measures such as hemoglobin, albumin, lactate dehydrogenase (LDH), leukocyte count, platelet count, renal function, and calcium may reveal distinct inflammatory–hematopoietic phenotypes even when median CRP values are similar.

We therefore assembled a large, single-center retrospective cohort spanning 21 hematologic disorders and analyzed pretreatment CRP distributions using a consistent clinical data framework. We performed prespecified within-category comparisons and CRP-quartile analyses to describe how rising CRP tracked with hematopoietic, nutritional, biochemical, and organ-function measures. We also used NHANES records as an unweighted exploratory comparison rather than a formal external validation cohort, recognizing that NHANES identifies a self-reported history of leukemia or lymphoma and does not distinguish active, newly diagnosed, or previously treated disease. Our objective was to define the contexts in which routine CRP measurement is informative and those in which it has limited discriminatory value.

## 2 Materials and methods

### 2.1 Study design and setting

This retrospective observational study included eligible inpatients and outpatients evaluated in the Department of Hematology at Nanjing Drum Tower Hospital between January 2009 and December 2023. The study was designed and reported in accordance with the principles of the Strengthening the Reporting of Observational Studies in Epidemiology (STROBE) statement (13). The primary analysis characterized baseline serum CRP across hematologic disorders; secondary analyses evaluated clinically relevant disease subtypes, correlations with routine laboratory variables, and gradients across CRP quartiles.

The study was approved by the Medical Ethics Committee of Nanjing Drum Tower Hospital, the Affiliated Hospital of Nanjing University Medical School (approval No. 2026-0668-01; June 23, 2026). The requirement for written informed consent was waived because the study used retrospective clinical data and involved no prospective intervention.

### 2.2 Participants and disease classification

Patients were eligible if they were aged ≥14 years, had a diagnosis established according to contemporaneous authoritative criteria, had a serum CRP measurement and required clinical laboratory data available at initial evaluation, and had samples obtained before chemotherapy, targeted therapy, immunotherapy, hematopoietic stem-cell transplantation conditioning, or other disease-directed treatment. Diagnoses made across the study period were reconciled to the 2016 or 2022 World Health Organization frameworks for myeloid and lymphoid neoplasms where applicable (14, 15). MM was classified using accepted International Myeloma Working Group criteria (16).

The 21 disease categories were acute leukemia, chronic leukemia, NHL, HL, MM, myelodysplastic syndromes, immune thrombocytopenia, aplastic anemia, HLH, hemophilia, autoimmune hemolytic anemia, polycythemia vera, Castleman disease, essential thrombocythemia, pure red-cell aplasia, TTP, plasma-cell leukemia, POEMS syndrome, Langerhans-cell histiocytosis, Evans syndrome, and paroxysmal nocturnal hemoglobinuria. For detailed subtype analyses, acute leukemia was restricted to AML and ALL, chronic leukemia to CML and CLL, lymphoma to HL and NHL, and MM was stratified by EMD status.

EMD was determined from computed tomography, positron-emission tomography/computed tomography, magnetic resonance imaging, and pathology reports when available. Patients were excluded for a documented acute bacterial or viral infection, major trauma, or major surgery within 4 weeks; a concurrent active non-hematologic malignancy; end-stage renal disease, decompensated cirrhosis, New York Heart Association class III–IV heart failure, or another severe organ disorder likely to dominate CRP levels; two or more concurrent hematologic disorders; use of prednisone-equivalent glucocorticoids >20 mg/day, immunosuppressants, cytotoxic therapy, or biologic therapy within 3 months; pregnancy or lactation; or insufficient baseline data.

Infection exclusion was based on documented clinical diagnoses recorded in the electronic medical record. Because infection adjudication was not performed prospectively or uniformly across the 15-year cohort, microbiological cultures, imaging findings, febrile neutropenia, and antibiotic exposure before baseline sampling were not systematically reviewed for all participants. Accordingly, occult or incompletely documented infection could not be fully excluded.

### 2.3 Clinical variables and CRP measurement

The first eligible pretreatment CRP result recorded at the initial disease evaluation was used. Other extracted variables included age, sex, hemoglobin, leukocyte count, platelet count, albumin, LDH, creatinine, alanine aminotransferase, aspartate aminotransferase, and, for MM, β2-microglobulin and calcium. Measurements were obtained from the hospital laboratory information system and linked to diagnosis and treatment timing using the electronic medical record. Serum CRP was measured using an immunoturbidimetric method throughout the study period, within the hospital clinical laboratory and according to standardized procedures in use at the time of testing. Because the cohort spans 2009–2023, complete historical records of individual analyzers, reagent manufacturers, and potential platform changes were not uniformly retrievable. Continuous variables were analyzed in their original units; CRP was expressed in mg/L and β2-microglobulin in ng/mL.

### 2.4 Subtype and CRP-quartile analyses

Four clinically prespecified comparisons were performed: AML versus ALL, CML versus CLL, HL versus NHL, and MM with versus without EMD. Within each subtype, patients were divided into disease-specific CRP quartiles. Disease-specific quartiles were selected as exploratory analytical strata because CRP distributions were skewed and differed substantially across hematologic disorders, allowing assessment of within-disease gradients without imposing a universal CRP cutoff across biologically heterogeneous conditions. These quartiles were not intended to define diagnostic, prognostic, or therapeutic decision thresholds. Quartile analyses were used to assess whether routine laboratory variables changed across increasing CRP strata. Spearman rank correlations were calculated between CRP and available continuous laboratory measures within each disease subtype.

### 2.5 Exploratory NHANES analysis

An exploratory analysis used six nonconsecutive NHANES cycles: 2003–2004, 2005–2006, 2007–2008, 2009–2010, 2015–2016, and 2017–2018 (17). A self-reported physician diagnosis recorded in questionnaire item MCQ230A was used to identify participants reporting leukemia (code 21) or lymphoma/Hodgkin disease (code 24). Participants reporting no cancer were used as the non-cancer comparison group. Extracted variables included high-sensitivity CRP (hs-CRP), hemoglobin, and albumin.

The NHANES component was analyzed using available unweighted records; complex-survey weights and age- or sex-adjusted models were not applied. Because NHANES does not establish active disease status, histologic subtype, time since diagnosis, relapse status, or treatment exposure, this analysis was interpreted as descriptive and hypothesis generating rather than as a population-representative estimate or formal external validation of the hospital cohort.

### 2.6 Statistical analysis

Analyses were performed using R version 4.3.1 (R Foundation for Statistical Computing, Vienna, Austria) and GraphPad Prism version 9.5.1 (GraphPad Software, San Diego, CA, United States). The ggplot2 and pheatmap packages were used for visualization. Normality was evaluated using the Shapiro–Wilk test. Normally distributed variables are summarized as mean ± standard deviation and were compared using independent-samples *t* tests or one-way analysis of variance with Bonferroni-adjusted *post hoc* testing. Non-normally distributed variables are summarized as median (interquartile range [IQR]) and were compared using Mann–Whitney *U* tests or Kruskal–Wallis tests with Dunn–Bonferroni *post hoc* testing. Categorical variables were compared using Pearson’s *χ²* test or Fisher’s exact test, as appropriate. Correlations were evaluated using Pearson or Spearman methods according to distributional assumptions. All tests were two-sided, and *P* < 0.05 was considered statistically significant.

Available-case analysis was used for variables with missing information. Sex percentages were calculated only among participants with documented sex; participants with unknown sex were excluded from sex-specific percentages and comparisons. No missing values were imputed. The cross-disease, subtype-specific correlation, and quartile analyses were exploratory. Nominal *P* values are reported for most correlation screens; therefore, individual associations, particularly those near the significance threshold, should be interpreted in the context of multiplicity, effect direction, biological plausibility, and consistency across analyses. Disease-specific stage, cytogenetic abnormalities, molecular risk classifications, and quantitative measures of disease burden were not consistently available across the 21 disorders and the extended study period; therefore, uniform multivariable models were not undertaken. The analyses were intended to be descriptive and hypothesis-generating, and between-group differences should not be interpreted as independent effects of disease subtype.

## 3 Results

### 3.1 CRP distribution across 21 hematologic disorders

The hospital cohort included 4,823 patients across 21 hematologic disease categories. Sex was documented for 4,295 patients, of whom 2,410 (56.1%) were male; 528 patients had unknown sex. Detailed cross-disease demographic and laboratory characteristics are provided in **Supplementary Table 1**. Baseline CRP differed markedly across disorders (global *P* < 0.001). HLH had the highest median CRP (44.6 mg/L; IQR, 18.5–82.0), followed by TTP (25.4 mg/L; IQR, 5.0–56.0) and acute leukemia (12.0 mg/L; IQR, 3.4–48.0). In contrast, median CRP was <4 mg/L in polycythemia vera, MM, Castleman disease, immune thrombocytopenia, essential thrombocythemia, pure red-cell aplasia, Langerhans-cell histiocytosis, and paroxysmal nocturnal hemoglobinuria. The wide within-disease distributions and substantial overlap between several disorders indicate that CRP concentration alone cannot classify hematologic disease. The corresponding cross-disease CRP distributions are shown in **Figure 1**.

**Figure 1:**
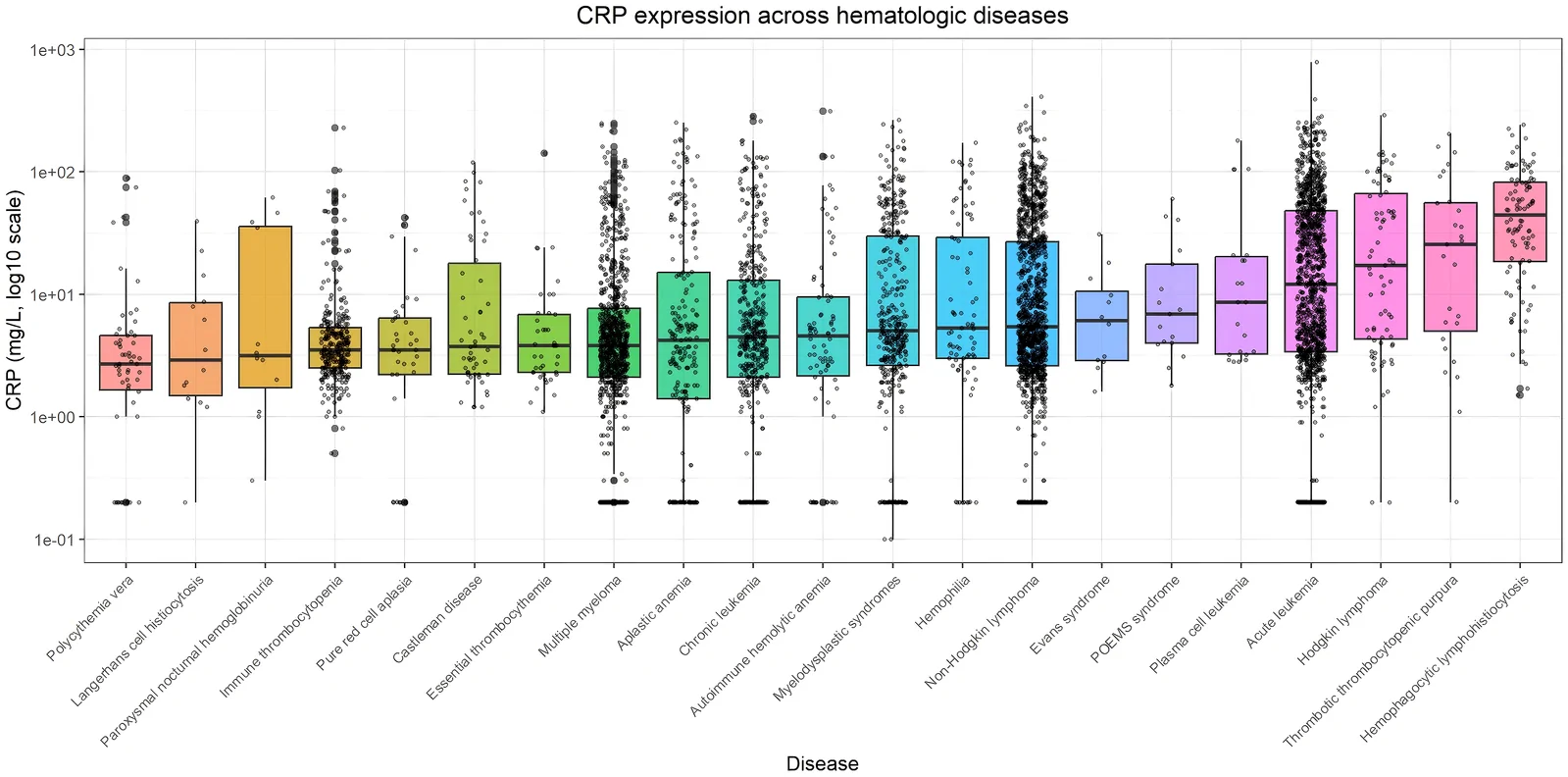
Baseline CRP distribution across 21 hematologic disorders. CRP is displayed on a log10 scale; points represent individual patients and boxes show medians and interquartile ranges. Marked between-disease heterogeneity is evident, with prominent elevations in HLH and TTP.

### 3.2 Acute leukemia: AML versus ALL

Subtype-level data were available for 758 patients with AML or ALL. Patients with AML were older than those with ALL (median, 63.0 vs 55.0 years; *P* < 0.001). Median CRP was higher in AML than in ALL (18.5 mg/L [IQR, 3.9–54.9] vs 9.2 mg/L [IQR, 3.2–45.6]; *P* = 0.004). AML was also associated with lower hemoglobin and albumin, whereas leukocyte count and LDH did not differ significantly between subtypes. Baseline demographic and laboratory comparisons between AML and ALL are summarized in **Table 1**. The corresponding distributions are shown in **Figure 2**.

**Table 1:** Baseline characteristics of patients with acute myeloid leukemia and acute lymphoblastic leukemia.

Characteristic	Total (*n* = 758)	AML (*n* = 333)	ALL (*n* = 425)	*P* value
Age, years	58.0 (43.0–70.0)	63.0 (49.0–72.0)	55.0 (37.0–69.0)	< 0.001
Male sex, *n* (%)	374 (49.3)	160 (48.0)	214 (50.4)	0.076
CRP, mg/L	12.5 (3.5–48.8)	18.5 (3.9–54.9)	9.2 (3.2–45.6)	0.004
Hemoglobin, g/L	81.0 (64.0–103.0)	76.0 (61.0–98.0)	83.0 (66.0–109.0)	< 0.001
WBC, ×10^9^/L	9.1 (3.2–37.5)	9.2 (2.9–36.9)	8.9 (3.4–37.7)	0.578
Platelet count, ×10^9^/L	48.0 (25.0–90.0)	43.0 (24.0–79.0)	50.0 (28.0–99.0)	0.056
Albumin, g/L	38.1 (34.7–40.6)	37.8 (34.5–40.1)	38.4 (35.0–41.0)	0.014
LDH, U/L	366.0 (220.0–680.8)	363.0 (229.0–631.0)	369.0 (215.0–740.0)	0.526

Data are presented as median (interquartile range) unless otherwise indicated. Sex percentages were calculated among participants with documented sex data. AML, acute myeloid leukemia; ALL, acute lymphoblastic leukemia; CRP, C-reactive protein; WBC, white blood cell count; LDH, lactate dehydrogenase.

**Figure 2:**
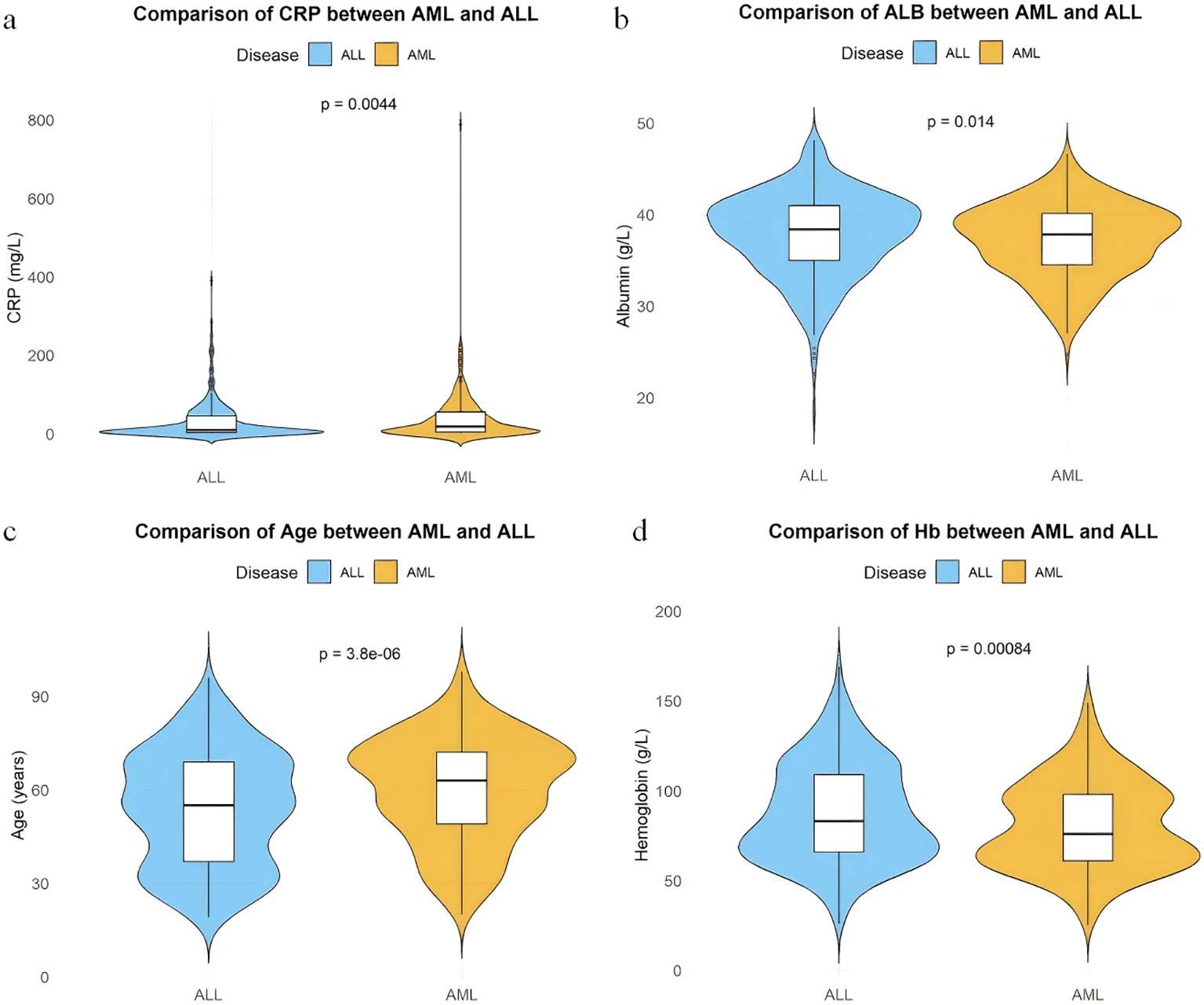
Distribution of **(A)** CRP, **(B)** albumin, **(C)** age, and **(D)** hemoglobin in AML and ALL. Violin plots show individual observations with summary statistics overlaid.

CRP showed different correlation patterns across the two subtypes. In AML, CRP correlated positively with age and inversely with albumin and hemoglobin, without significant correlations with LDH or platelet count. In ALL, CRP correlated positively with age and LDH and inversely with albumin, hemoglobin, and platelet count. These subtype-specific correlations are shown in **Figure 3**.

**Figure 3:**
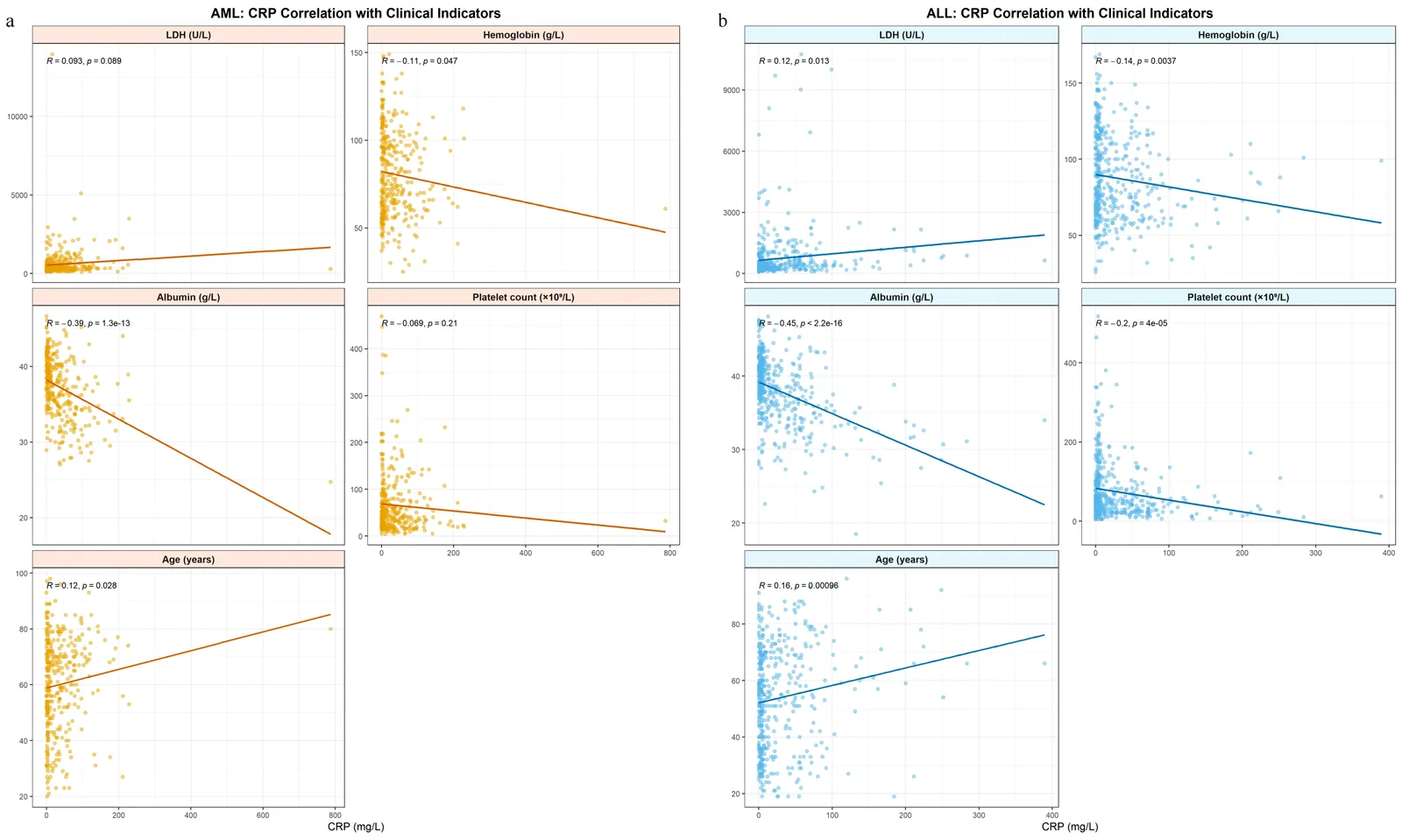
Subtype-specific Spearman correlations between CRP and clinical laboratory variables in AML **(A)** and ALL **(B)**. The direction and statistical strength of associations differ between the two acute leukemia subtypes.

Quartile analyses reinforced the association between CRP and a broader systemic illness phenotype. In AML, LDH increased from 288 U/L in Q1 to 451 U/L in Q4 (*P* = 0.039), while albumin decreased from 39.8 to 34.3 g/L (*P* < 0.001); hemoglobin and platelet count showed borderline downward trends. In ALL, increasing CRP was accompanied by higher LDH (265 to 566.5 U/L; *P* = 0.0002), lower albumin (40.1 to 34.7 g/L; *P* < 0.0001), lower platelet count (62 to 37×10^9^/L; *P* < 0.0001), lower hemoglobin (81 to 77 g/L; *P* = 0.0008), and older age (55.0 to 60.5 years; *P* = 0.0081). Detailed quartile-stratified results for AML and ALL are presented in **Tables 2** and **3**, respectively.

**Table 2:** Clinical and laboratory characteristics across C-reactive protein quartiles in acute myeloid leukemia.

Group	*N*	Age (years)	Albumin (g/L)	Hemoglobin (g/L)	LDH (U/L)	Platelet count (×10^9^/L)
Q1 (Lowest CRP)	85	56 (43-72)	39.8 (38.1-41.4)	76 (58-108)	288 (185-511)	50 (27-92)
Q2 (Low CRP)	82	62.5 (49.5-72)	38.4 (36.2-39.9)	85 (62-101)	301.5 (212.2-508)	42.5 (23-73.8)
Q3 (High CRP)	84	62.5 (44.8-70)	36.4 (34-39.9)	79 (65.8-96.2)	413 (270.5-672.5)	48.5 (27-80)
Q4 (Highest CRP)	82	67 (53.5-74)	34.3 (32.1-37)	70 (60.2-93.5)	451 (292-987)	37 (20-76.8)
*P*		0.079	< 0.001	0.051	0.039	0.066

Data are presented as median (interquartile range). *P* values compare the four disease-specific C-reactive protein quartiles.

**Table 3:** Clinical and laboratory characteristics across C-reactive protein quartiles in acute lymphoblastic leukemia.

Group	*N*	Age (years)	Albumin (g/L)	Hemoglobin (g/L)	LDH (U/L)	Platelet count (×10^9^/L)
Q1 (Lowest CRP)	109	55 (36-66)	40.1 (38.1-41.9)	81 (66-115)	265 (182-518)	62 (33-108)
Q2 (Low CRP)	105	49 (36-64)	40.2 (37.8-41.8)	94 (73-118)	254 (184-536)	67 (36-134)
Q3 (High CRP)	105	56 (40-70)	37.4 (34.7-39.7)	79 (63-102)	427 (271-1033)	46 (25-78)
Q4 (Highest CRP)	106	60.5 (42-73.8)	34.7 (32.1-38.1)	77 (61.2-96.5)	566.5 (353.2-1064.2)	37 (22-61.8)
*P*		0.0081	< 0.0001	0.0008	0.0002	< 0.0001

Data are presented as median (interquartile range). *P* values compare the four disease-specific C-reactive protein quartiles.

### 3.3 Chronic leukemia: CML versus CLL

The CML–CLL analysis included 367 patients (279 with CML and 88 with CLL). CML patients were younger than CLL patients (55.0 vs 73.0 years; *P* < 0.001), but median CRP did not differ significantly (4.7 mg/L [IQR, 2.2–12.5] vs 3.8 mg/L [IQR, 1.8–8.7]; *P* = 0.150). As expected from the underlying diseases, CML was associated with higher leukocyte count, platelet count, and LDH and lower hemoglobin. Baseline characteristics of the CML and CLL groups are summarized in **Table 4**. The corresponding distributions are shown in **Figure 4**.

**Table 4:** Baseline characteristics of patients with chronic myeloid leukemia and chronic lymphocytic leukemia.

Characteristic	Total (*n* = 367)	CML (*n* = 279)	CLL (*n* = 88)	*P* value
Age, years	60.0 (46.0–72.0)	55.0 (40.5–69.0)	73.0 (66.0–80.0)	< 0.001
Male sex, *n* (%)	197 (53.7)	143 (51.3)	54 (61.4)	0.202
Laboratory parameters				
CRP, mg/L	4.3 (2.1–12.4)	4.7 (2.2–12.5)	3.8 (1.8–8.7)	0.150
Hemoglobin, g/L	105.0 (85.0–123.0)	101.0 (83.0–120.0)	116.5 (93.0–133.0)	< 0.001
White blood cell count, ×10^9^/L	48.7 (14.4–129.8)	66.3 (13.2–147.5)	24.5 (15.6–51.3)	0.001
Platelet count, ×10^9^/L	197.0 (112.5–414.5)	242.0 (125.0–530.5)	134.5 (91.8–168.2)	< 0.001
Albumin, g/L	39.7 (37.1–42.5)	39.9 (37.0–42.5)	39.3 (37.4–42.1)	0.445
LDH, U/L	482.0 (223.0–830.5)	642.0 (332.0–902.0)	206.0 (170.2–274.5)	< 0.001

Data are presented as median (interquartile range) unless otherwise indicated. Sex percentages were calculated among participants with documented sex data. CML, chronic myeloid leukemia; CLL, chronic lymphocytic leukemia; CRP, C-reactive protein; LDH, lactate dehydrogenase.

**Figure 4:**
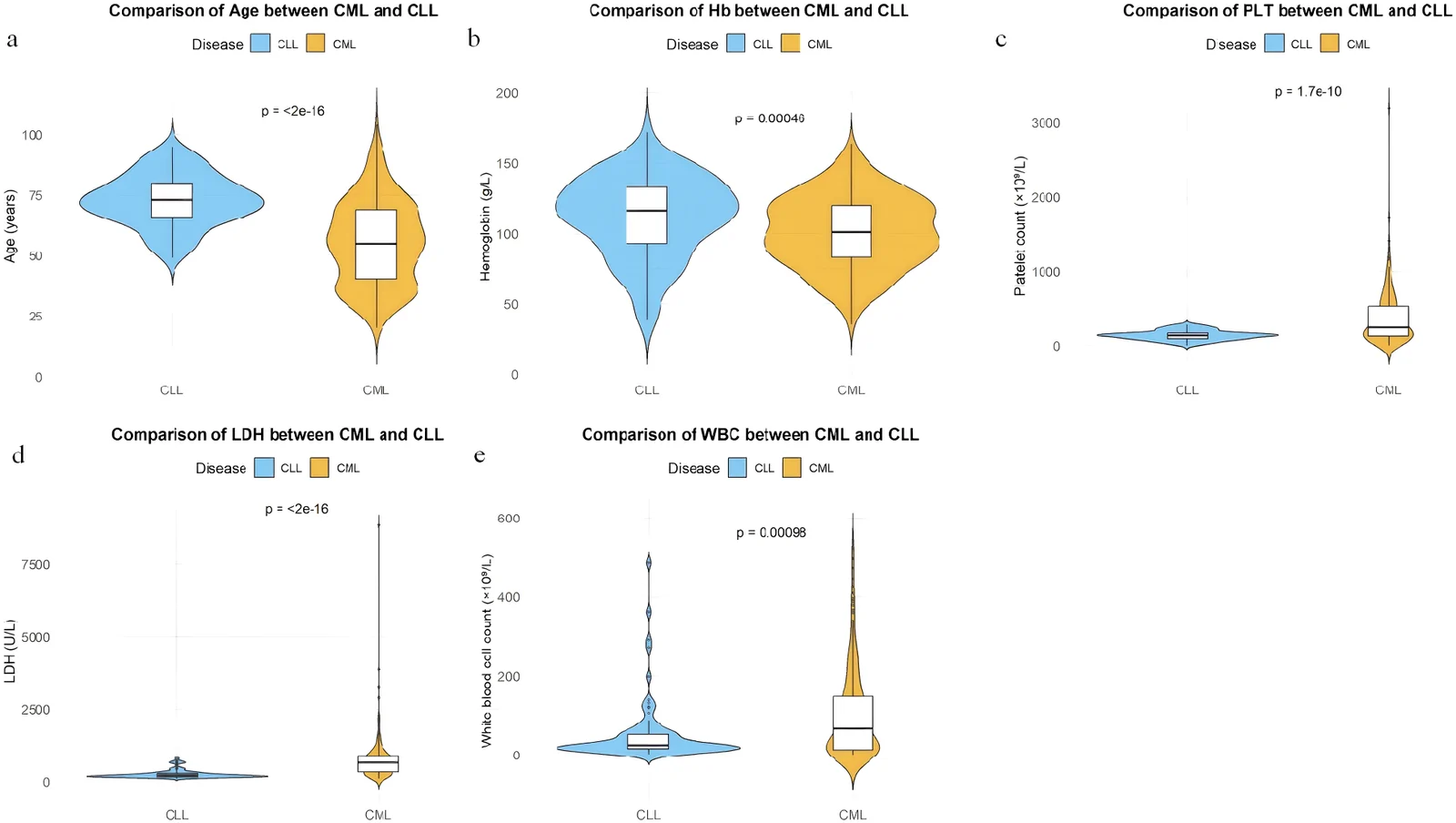
Distribution of **(A)** age, **(B)** hemoglobin, **(C)** platelet count, **(D)** LDH, and **(E)** white blood cell count in CML and CLL. Despite similar CRP distributions, the hematologic and biochemical phenotypes differ substantially between subtypes.

In CML, CRP correlated positively with age and LDH and inversely with albumin, hemoglobin, and platelet count. In CLL, significant inverse associations were limited to albumin and hemoglobin; CRP was not significantly associated with age, platelet count, or LDH. These subtype-specific correlations are shown in **Figure 5**. Consistent with these correlations, CML quartiles showed progressively higher LDH and lower hemoglobin and albumin (all *P* < 0.0001), whereas the clearest gradient in CLL was a decline in albumin (*P* = 0.0053). Thus, similar median CRP concentrations concealed distinct accompanying laboratory phenotypes. The corresponding CRP-quartile analyses for CML and CLL are detailed in **Tables 5** and **6**, respectively.

**Figure 5:**
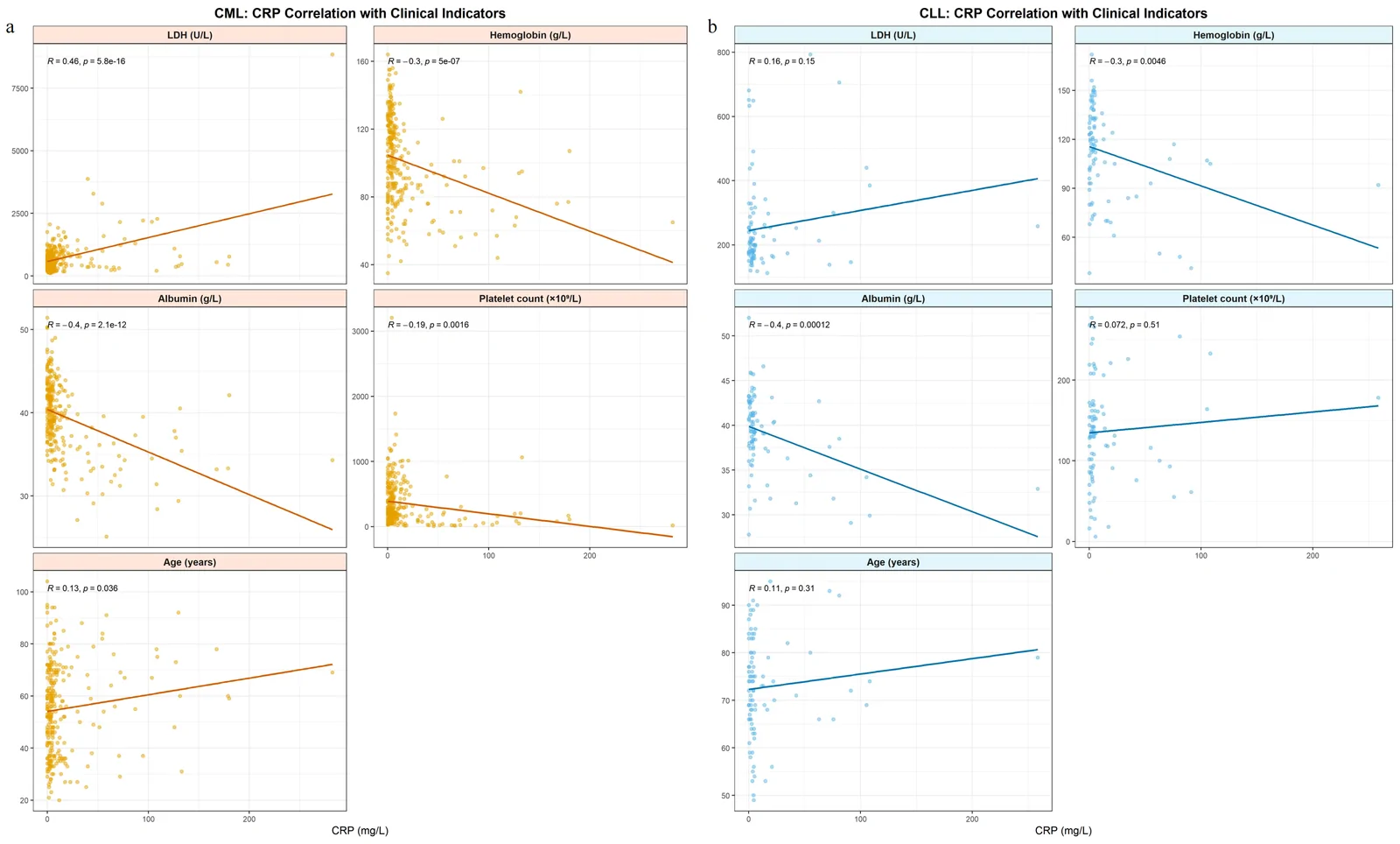
Subtype-specific Spearman correlations between CRP and clinical laboratory variables in CML **(A)** and CLL **(B)**. CML demonstrates a broader CRP-associated laboratory profile than CLL.

**Table 5:** Clinical and laboratory characteristics across C-reactive protein quartiles in chronic myeloid leukemia.

Group	*N*	LDH (U/L)	Hemoglobin (g/L)	Platelet count (×10^9^/L)	Albumin (g/L)	Age (years)
Q1 (Lowest CRP)	70	604 (277.8-808.5)	109 (84.5-128)	288 (194.8-596)	41.8 (39.3-43.6)	52.5 (42-62)
Q2 (Low CRP)	70	551.5 (306.2-836.5)	114 (98.2-127.5)	238 (128.2-500.2)	40.8 (38.4-42.6)	54.5 (38.2-66)
Q3 (High CRP)	69	698 (310-906)	102 (87-118)	273 (169-514)	39.6 (37.9-42.6)	59 (44-69)
Q4 (Highest CRP)	70	781 (429.2-1171.2)	84.5 (66-94.8)	163 (49.5-497)	36 (33.2-39.6)	57 (37-71)
*P*		< 0.0001	< 0.0001	0.1015	< 0.0001	0.2275

Data are presented as median (interquartile range). *P* values compare the four disease-specific C-reactive protein quartiles.

**Table 6:** Clinical and laboratory characteristics across C-reactive protein quartiles in chronic lymphocytic leukemia.

Group	*N*	LDH (U/L)	Hemoglobin (g/L)	Platelet count (×10^9^/L)	Albumin (g/L)	Age (years)
Q1 (Lowest CRP)	23	211 (180-271)	114 (94.5-124)	128 (85-161)	41.3 (37.9-42.7)	74 (68-78.5)
Q2 (Low CRP)	22	178 (157-218.2)	135.5 (114-145.5)	136.5 (93.8-160)	40.1 (38.2-41.4)	72 (65.2-78.8)
Q3 (High CRP)	21	200 (181-299)	127 (112-138)	152 (130-169)	39.3 (38.4-41.1)	72 (63-83)
Q4 (Highest CRP)	22	239.5 (163-299.2)	92.5 (70-107.8)	126 (94.8-175.2)	37.2 (33-39.7)	73 (69-79)
*P*		0.7214	0.0509	0.4875	0.0053	0.8923

Data are presented as median (interquartile range). *P* values compare the four disease-specific C-reactive protein quartiles.

### 3.4 Lymphoma: HL versus NHL

The lymphoma comparison included 75 patients with HL and 1,127 with NHL. HL patients were substantially younger (45.0 vs 64.0 years; *P* < 0.001) and had higher median CRP (17.2 mg/L [IQR, 4.3–66.2] vs 5.4 mg/L [IQR, 2.6–26.9]; *P* < 0.001). Leukocyte and platelet counts were higher in HL, whereas hemoglobin, albumin, and LDH did not differ significantly between the broad lymphoma categories. Baseline characteristics of the HL and NHL groups are shown in **Table 7**.

**Table 7:** Baseline characteristics of patients with Hodgkin lymphoma and non-Hodgkin lymphoma.

Characteristic	Total (*n* = 1202)	HL (*n* = 75)	NHL (*n* = 1127)	*P* value
Age, years	63.0 (52.8–74.0)	45.0 (32.0–68.0)	64.0 (53.0–74.0)	< 0.001
Male sex, *n* (%)	622 (51.7)	37 (49.3)	585 (51.9)	0.049
Laboratory parameters				
CRP, mg/L	5.6 (2.6–28.3)	17.2 (4.3–66.2)	5.4 (2.6–26.9)	< 0.001
Hemoglobin, g/L	122.0 (104.0–137.0)	122.0 (105.5–136.0)	122.0 (104.0–137.0)	0.924
White blood cell count, ×10^9^/L	5.7 (4.3–7.5)	7.1 (4.5–11.0)	5.7 (4.3–7.4)	0.001
Platelet count, ×10^9^/L	190.0 (140.0–247.0)	248.0 (184.5–324.5)	188.0 (139.0–243.0)	< 0.001
Albumin, g/L	38.2 (34.9–40.9)	39.4 (36.5–41.2)	38.2 (34.8–40.9)	0.215
LDH, U/L	213.0 (169.2–323.8)	228.0 (183.5–297.5)	211.0 (168.5–325.0)	0.324

Data are presented as median (interquartile range) unless otherwise indicated. Sex percentages were calculated among participants with documented sex data. HL, Hodgkin lymphoma; NHL, non-Hodgkin lymphoma; CRP, C-reactive protein; LDH, lactate dehydrogenase.

CRP correlated positively with leukocyte count, platelet count, and LDH and inversely with hemoglobin and albumin in HL. In NHL, CRP correlated positively with platelet count and LDH and inversely with hemoglobin and albumin, without significant associations with age or leukocyte count. These subtype-specific correlations are shown in **Figure 6**. Across HL quartiles, CRP was accompanied by increasing LDH, leukocyte count, and platelet count and decreasing hemoglobin and albumin. Across NHL quartiles, LDH and leukocyte count increased and hemoglobin and albumin decreased, while the statistically significant platelet difference was non-monotonic. Detailed CRP-quartile results for HL and NHL are provided in **Tables 8** and **9**, respectively.

**Table 8:** Clinical and laboratory characteristics across C-reactive protein quartiles in Hodgkin lymphoma.

Group	*N*	LDH (U/L)	Hemoglobin (g/L)	Albumin (g/L)	Platelet count (×10^9^/L)	White blood cell count (×10^9^/L)	Age (years)
Q1 (Lowest CRP)	19	208 (173-229.5)	127 (121-148.5)	40.6 (39.5-41.6)	215 (169.5-293.5)	5.4 (4.1-6.9)	43 (32-61)
Q2 (Low CRP)	19	206 (168-249.5)	124 (119-141.5)	40.2 (38.2-41.5)	214 (175-271)	7.3 (5-10.2)	48 (30.5-68.5)
Q3 (High CRP)	18	249.5 (216-526)	117 (104.8-134)	39.4 (36.5-41)	260.5 (210.5-328)	7.4 (5.1-11.8)	37 (29.8-64)
Q4 (Highest CRP)	19	271 (221-378.5)	109 (87.5-125.5)	32.1 (29.4-37.7)	331 (229-410.5)	11.2 (5.4-13.6)	61 (34.5-78)
*P*		0.0027	< 0.0001	< 0.0001	0.0068	0.0017	0.1221

Data are presented as median (interquartile range). *P* values compare the four disease-specific C-reactive protein quartiles.

**Table 9:** Clinical and laboratory characteristics across C-reactive protein quartiles in non-Hodgkin lymphoma.

Group	*N*	LDH (U/L)	Hemoglobin (g/L)	Albumin (g/L)	Platelet count (×10^9^/L)	White blood cell count (×10^9^/L)	Age (years)
Q1 (Lowest CRP)	294	177 (152.2-216)	130 (117-144)	40.3 (37.9-42.6)	185 (145-237)	5.3 (4.3-6.6)	63 (54-74)
Q2 (Low CRP)	274	192 (163-240)	130 (117-141)	39.7 (37.6-41.5)	188.5 (148-232.8)	5.5 (4.3-6.6)	62 (53-72)
Q3 (High CRP)	277	233 (183-344)	121 (105-134)	37.8 (35.2-40.4)	184 (136-239)	5.6 (4.1-7.6)	66 (54-74)
Q4 (Highest CRP)	282	335 (222.2-622.5)	105 (85-122)	33.6 (29.9-36.8)	190 (126-276)	6.8 (4.8-9.2)	65 (53-75)
*P*		< 0.0001	< 0.0001	< 0.0001	0.0193	0.0015	0.7582

Data are presented as median (interquartile range). *P* values compare the four disease-specific C-reactive protein quartiles.

**Figure 6:**
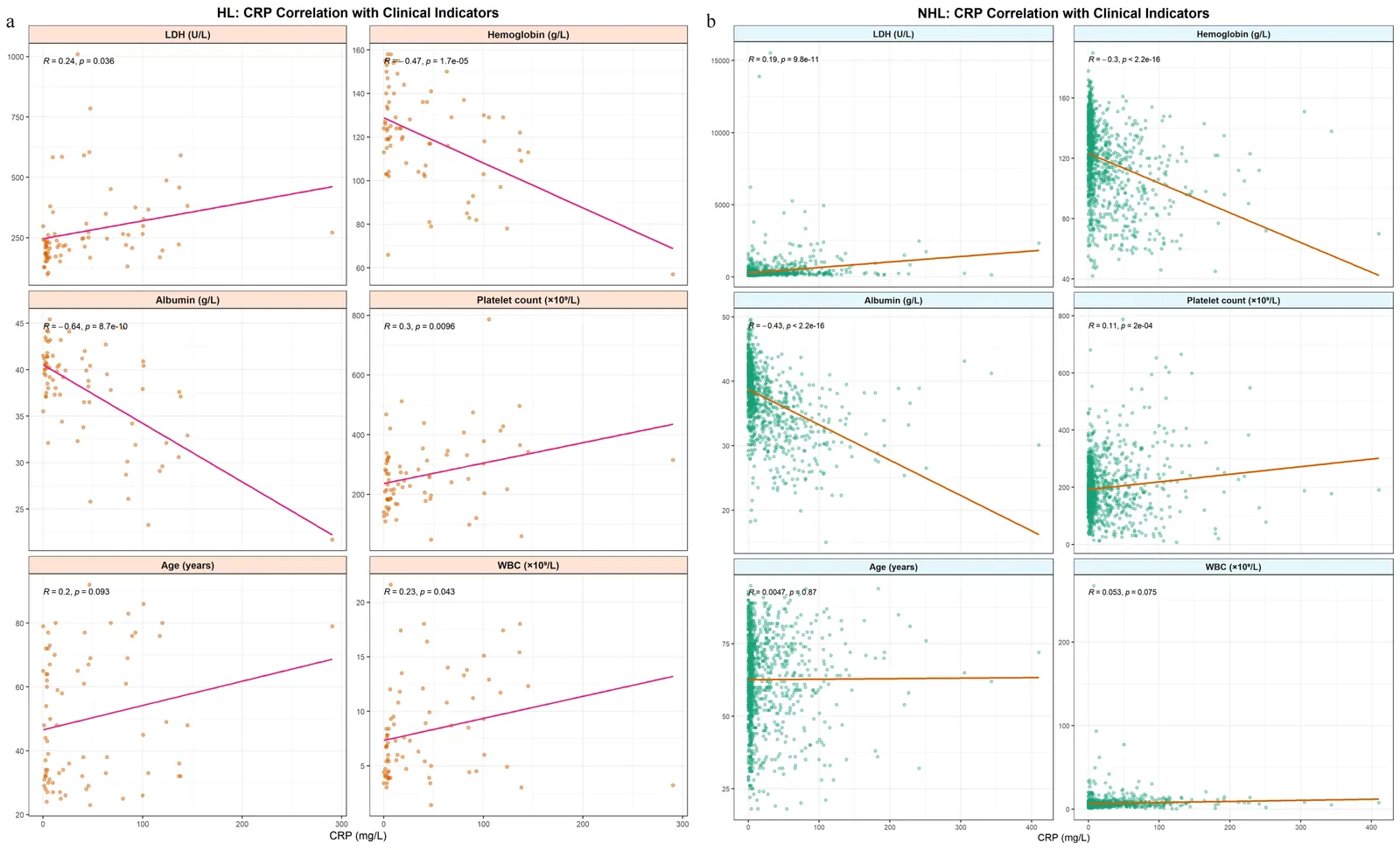
Spearman correlations between CRP and clinical laboratory variables in HL **(A)** and NHL **(B)**. Positive leukocyte and platelet associations are more prominent in HL.

### 3.5 Multiple myeloma: patients with versus without EMD

Among 754 patients with MM, 78 had EMD and 676 did not. Patients with EMD were younger (59.5 vs 69.0 years; *P* < 0.001), but CRP distributions were nearly identical (median 3.8 mg/L in both groups; *P* = 0.712). The corresponding CRP distributions are shown in **Figure 7**. Patients with EMD had higher hemoglobin and platelet counts and lower LDH, while leukocyte count, albumin, β2-microglobulin, creatinine, and calcium did not differ significantly. These differences likely reflect cohort composition and cannot be interpreted as effects of EMD without multivariable adjustment. Baseline comparisons between MM patients with and without EMD are summarized in **Table 10**.

**Figure 7:**
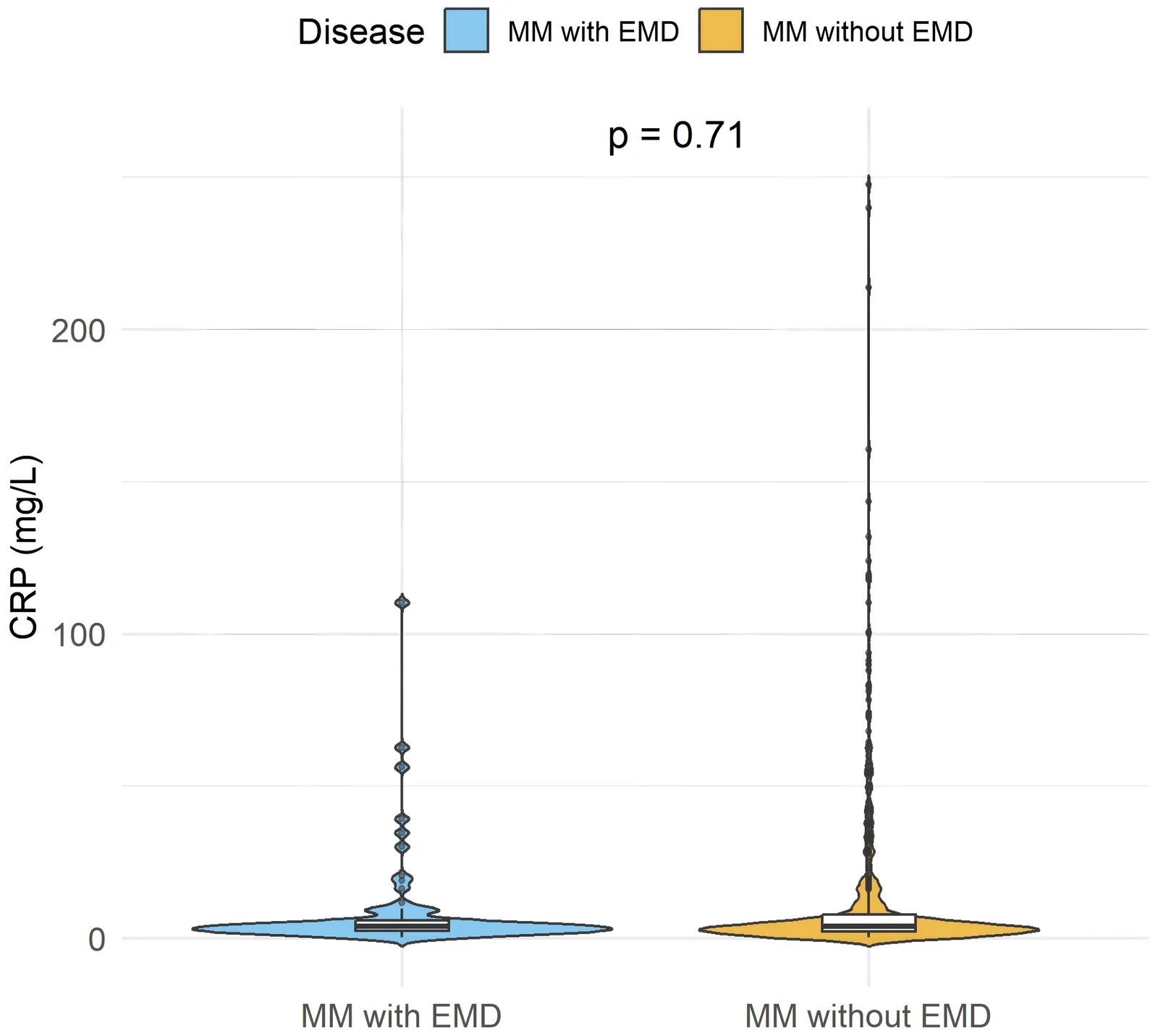
CRP distribution in MM with and without EMD. The two distributions overlap extensively, and median CRP does not differ between groups (*P* = 0.712).

**Table 10:** Baseline characteristics of patients with multiple myeloma with and without extramedullary disease.

Characteristic	Total (*n* = 754)	MM with EMD (*n* = 78)	MM without EMD (*n* = 676)	*P* value
Age, years	68.0 (59.0–75.0)	59.5 (53.2–65.0)	69.0 (60.0–76.0)	< 0.001
Male sex, *n* (%)	384 (50.9)	47 (60.3)	337 (49.9)	0.017
Laboratory parameters				
CRP, mg/L	3.8 (2.1–7.7)	3.8 (2.3–5.8)	3.8 (2.1–7.7)	0.712
Hemoglobin, g/L	88.0 (70.2–109.0)	97.0 (74.8–115.5)	87.0 (69.0–108.0)	0.016
White blood cell count, ×10^9^/L	4.6 (3.5–6.0)	4.8 (4.0–6.3)	4.6 (3.5–6.0)	0.165
Platelet count, ×10^9^/L	153.0 (106.0–207.0)	172.5 (136.2–214.2)	151.0 (103.8–206.2)	0.016
Albumin, g/L	34.1 (29.6–39.0)	35.9 (30.9–40.2)	33.9 (29.5–39.0)	0.105
LDH, U/L	176.0 (141.2–230.0)	162.0 (134.5–189.8)	178.0 (142.8–233.0)	0.008
β2-microglobulin, mg/L	3901.5 (2618.2–7158.8)	3371.5 (2574.5–6367.8)	4005.0 (2668.5–7211.5)	0.231
Creatinine, μmol/L	81.0 (58.8–137.0)	72.0 (52.2–109.5)	83.0 (59.0–141.6)	0.054
Calcium, mmol/L	2.4 (2.2–2.6)	2.4 (2.3–2.6)	2.4 (2.2–2.6)	0.214

Data are presented as median (interquartile range) unless otherwise indicated. Sex percentages were calculated among participants with documented sex data. MM, multiple myeloma; EMD, extramedullary disease; CRP, C-reactive protein; LDH, lactate dehydrogenase.

In MM with EMD, CRP was not significantly associated with age, blood counts, albumin, LDH, β2-microglobulin, creatinine, or calcium. In MM without EMD, CRP correlated positively with leukocyte count and LDH and inversely with albumin. These subgroup-specific correlations are shown in **Figure 8**. No variable differed significantly across CRP quartiles in the EMD subgroup. In MM without EMD, rising CRP was accompanied by lower albumin, higher LDH, higher creatinine, and higher leukocyte count. These findings support CRP as a marker of systemic inflammatory and metabolic disturbance in selected MM patients but not as a marker of EMD itself. The CRP-quartile analyses for MM with and without EMD are presented in **Tables 11** and **12**, respectively.

**Figure 8:**
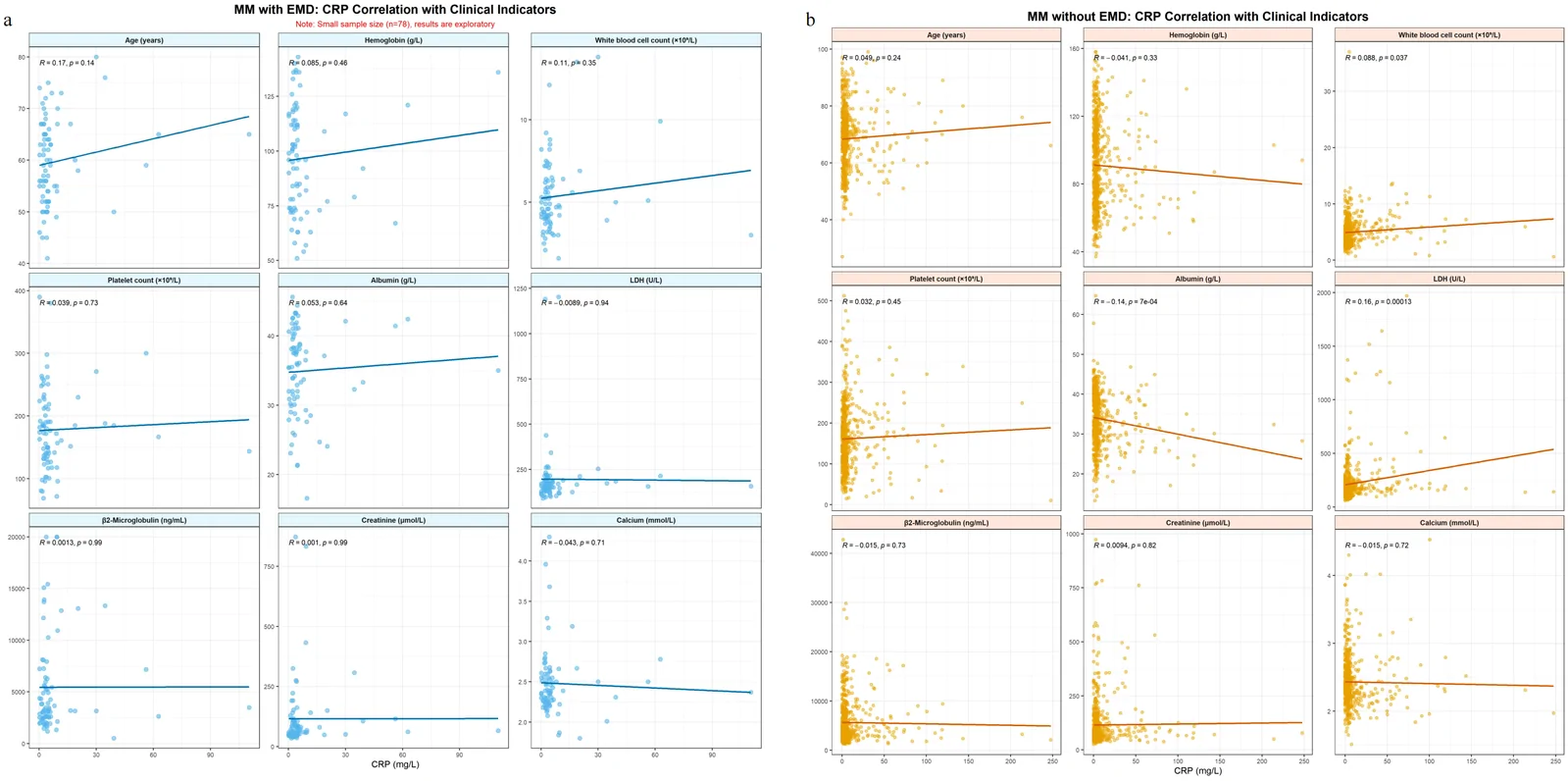
Spearman correlations between CRP and clinical laboratory variables in MM with EMD **(A)** and without EMD **(B)**. Significant associations with leukocyte count, LDH, and albumin are present only in the non-EMD subgroup.

**Table 11:** Clinical and laboratory characteristics across C-reactive protein quartiles in multiple myeloma with extramedullary disease.

Group	*N*	LDH (U/L)	Hemoglobin (g/L)	Albumin (g/L)	Platelet count (×10^9^/L)	Age (years)	White blood cell count (×10^9^/L)	β2-Microglobulin (ng/mL)	Creatinine (μmol/L)	Calcium (mmol/L)
Q1	20	148.5 (126-175)	97.5 (77-103)	34.6 (30.1-39.7)	183.5 (144-234.5)	56 (52.8-61.5)	4.3 (3.6-5.2)	3438 (2496.8-5472.2)	57 (48.2-90.2)	2.4 (2.3-2.6)
Q2	19	184 (145-220.5)	110 (76-118.5)	37.5 (31.2-40.6)	172 (134.5-206.5)	65 (57-67)	5.3 (4.1-6.3)	3460 (2311.5-6183)	97 (61.5-114.5)	2.3 (2.2-2.6)
Q3	19	156 (138-173)	111 (88.5-121)	37.3 (32.5-41)	150 (138.5-215)	54 (51.5-61)	5.2 (4-7.2)	3144 (2558-5094.5)	74 (51-86.5)	2.4 (2.3-2.6)
Q4	20	172 (150.8-189.5)	81 (71.2-99.2)	34.4 (29.1-38.2)	164 (138.2-188.8)	63 (57.2-67.8)	4.9 (3.7-6.3)	3577.5 (2957.8-11408.2)	70.5 (60.8-121.5)	2.4 (2.2-2.5)
*P*		0.8499	0.4721	0.6698	0.8067	0.2291	0.0822	0.2435	0.2982	0.7349

Data are presented as median (interquartile range). *P* values compare the four disease-specific C-reactive protein quartiles. EMD, extramedullary disease.

**Table 12:** Clinical and laboratory characteristics across C-reactive protein quartiles in multiple myeloma without extramedullary disease.

Group	*N*	Albumin (g/L)	LDH (U/L)	Hemoglobin (g/L)	White blood cell count (×10^9^/L)	Creatinine (μmol/L)	Platelet count (×10^9^/L)	Age (years)	β2-Microglobulin (ng/mL)	Calcium (mmol/L)
Q1 (Lowest CRP)	176	36.5 (31.1-40.3)	168.5 (133.8-221)	88 (69-108.2)	4.3 (3.1-5.7)	81 (57-116.2)	145.5 (105.8-197.5)	69 (61-77)	3881 (2591-6985)	2.4 (2.3-2.6)
Q2 (Low CRP)	166	34.9 (29.6-39.5)	179.5 (148-226)	92.5 (73-114)	4.6 (3.7-6)	74 (56.6-120.2)	157 (101-211.8)	66 (59-73)	3645.5 (2467-7232)	2.4 (2.2-2.6)
Q3 (High CRP)	166	33.7 (29.4-37.8)	174.5 (139-231.8)	83.5 (68-106)	4.5 (3.3-5.8)	85 (61.2-164)	140 (100-191.8)	70 (63.2-76)	4157 (2775-6966.8)	2.4 (2.2-2.6)
Q4 (Highest CRP)	168	32 (28.2-36)	192.5 (158-261)	83 (69-102.2)	5.2 (3.8-6.8)	91 (64-163.5)	163 (107-218.8)	70 (63-77)	4400 (2948-7692)	2.3 (2.1-2.5)
*P*		< 0.0001	< 0.0001	0.0605	0.0232	0.0019	0.2112	0.1319	0.4472	0.1351

Data are presented as median (interquartile range). *P* values compare the four disease-specific C-reactive protein quartiles. EMD, extramedullary disease.

### 3.6 Exploratory NHANES analysis

The unweighted NHANES analysis included 40 participants reporting leukemia, 71 reporting lymphoma, and 60,270 non-cancer participants. Median hs-CRP was 7.6 mg/L (IQR, 2.7–25.1) in the leukemia group, 3.8 mg/L (IQR, 1.7–23.0) in the lymphoma group, and 2.7 mg/L (IQR, 0.7–9.4) in the non-cancer group. The corresponding hs-CRP distributions are shown in **Figure 9**. However, the non-cancer group was much younger (median age, 25.0 years) than the leukemia and lymphoma groups (63.0 and 60.0 years), and the disease groups represented self-reported histories rather than uniformly active or untreated malignancy. Accordingly, the between-group pattern is descriptive and potentially confounded. Baseline characteristics of the exploratory NHANES groups are presented in **Table 13**.

**Figure 9:**
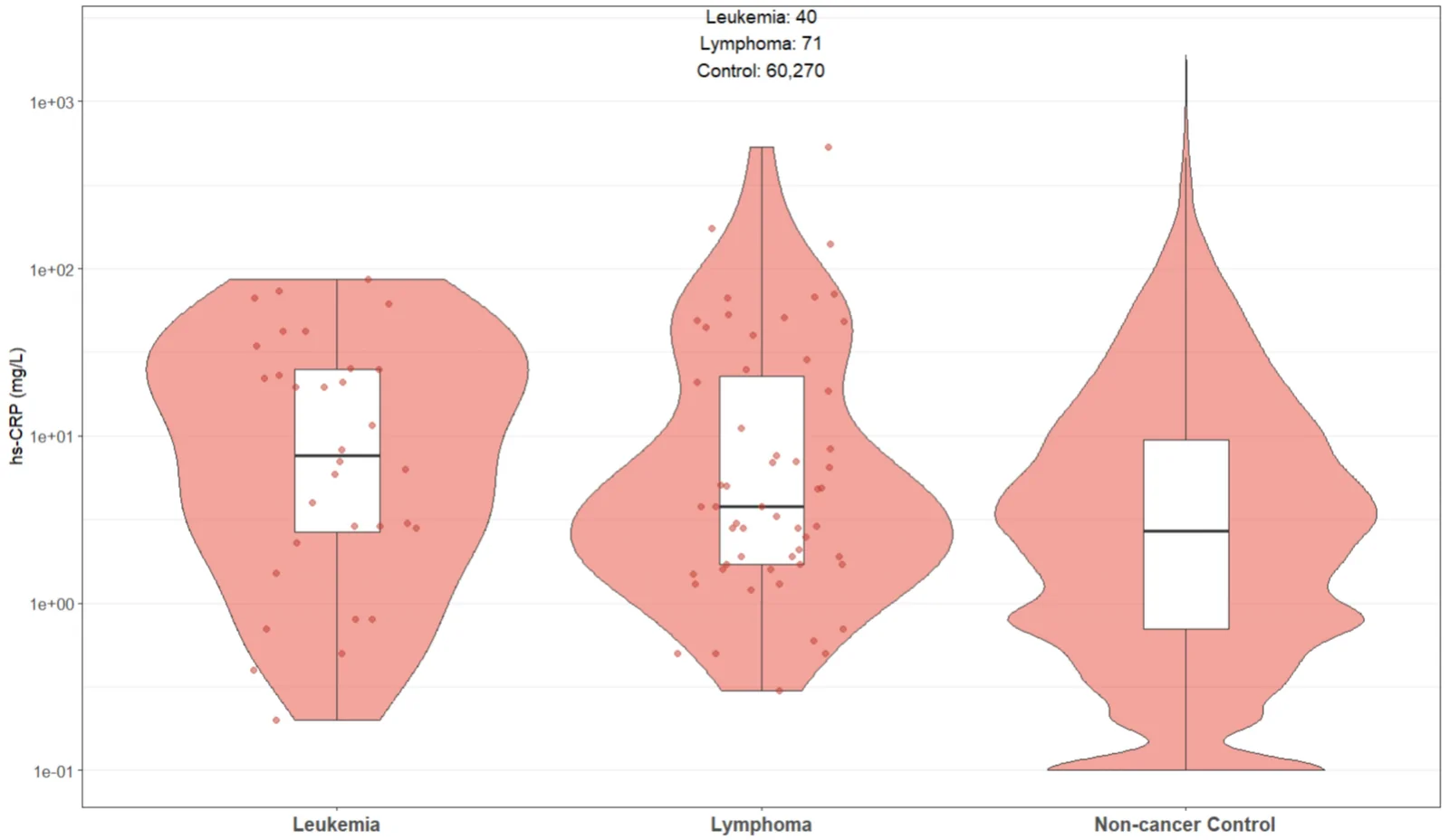
Unweighted distribution of hs-CRP among NHANES participants reporting leukemia, participants reporting lymphoma, and non-cancer participants. The plot is descriptive; interpretation is limited by self-reported disease history and major between-group age differences.

**Table 13:** Baseline characteristics of the unweighted exploratory NHANES sample.

Characteristic	Leukemia (*n* = 40)	Lymphoma (*n* = 71)	Non-cancer control (*n* = 60,270)
Age, years	63.0 (52.5–72.0)	60.0 (48.5–70.0)	25.0 (14.0–43.0)
Male sex, *n* (%)	22 (55.0)	38 (53.5)	29,472 (48.9)
hs-CRP, mg/L	7.6 (2.7–25.1)	3.8 (1.7–23.0)	2.7 (0.7–9.4)
Hemoglobin, g/dL	14.2 (12.2–15.0)	13.8 (12.1–14.8)	13.7 (12.6–14.8)
Albumin, g/dL	4.2 (3.9–4.5)	4.1 (3.9–4.4)	4.2 (3.9–4.5)

Data are presented as median (interquartile range) for continuous variables and *n* (%) for categorical variables. The exploratory sample was derived from six nonconsecutive NHANES cycles (2003–2004, 2005–2006, 2007–2008, 2009–2010, 2015–2016, and 2017–2018). Diagnoses were identified using the self-reported questionnaire item MCQ230A. hs-CRP, high-sensitivity C-reactive protein.

Within the combined leukemia and lymphoma group, hs-CRP correlated inversely with hemoglobin (*r*=−0.25; *P* = 0.003) and albumin (*r*=−0.33; *P* < 0.001). Directionally similar inverse associations were observed in the leukemia and lymphoma groups separately. These results are consistent with an association between higher systemic inflammation and anemia or lower albumin, but they do not establish that the relationship is specific to hematologic malignancy or independent of age, comorbidity, treatment, or disease activity. Group-specific and combined correlation coefficients are reported in **Table 14**.

**Table 14:** Spearman correlations between high-sensitivity C-reactive protein and hemoglobin or albumin in unweighted NHANES participants reporting leukemia or lymphoma.

Variable	Cohort	*n*	*r*	*P* value
CRP vs Hemoglobin	Leukemia	40	–0.31	0.007
	Lymphoma	71	–0.18	0.045
	Combined	111	–0.25	0.003
CRP vs Albumin	Leukemia	40	–0.38	< 0.001
	Lymphoma	71	–0.29	0.006
	Combined	111	–0.33	< 0.001

The combined group includes all participants reporting leukemia or lymphoma. *P* values were calculated using Spearman rank correlation. hs-CRP, high-sensitivity C-reactive protein.

## 4 Discussion

In this large retrospective cohort, pretreatment CRP varied substantially across 21 hematologic disorders and carried different accompanying laboratory signatures within clinically related disease groups. The most prominent CRP elevations occurred in HLH and TTP, while several chronic clonal, marrow-failure, immune, and plasma-cell disorders had comparatively low median values. Within neoplastic categories, AML had higher CRP than ALL, HL had higher CRP than NHL, and CML and CLL had similar median CRP despite markedly different correlation profiles. Most importantly, CRP did not differ between MM with and without EMD. The central implication is not that CRP distinguishes hematologic diagnoses, but that its meaning is conditioned by the underlying biology and by the broader hematologic, nutritional, biochemical, and organ-function phenotype.

The cross-disease results are biologically plausible because systemic CRP integrates several processes that vary across hematologic disorders: cytokine release, endothelial and tissue injury, tumor-cell turnover, marrow dysfunction, disease-related inflammatory activity, and nutritional or hepatic responses (1–4, 8, 11). HLH is characterized by uncontrolled immune-cell activation and cytokine excess (18), while TTP involves microvascular endothelial injury and systemic inflammatory signaling. Their high median CRP values therefore fit a hyperinflammatory phenotype. Nevertheless, the broad distributions in **Figure 1** demonstrate that CRP is non-specific. Very high CRP should prompt a systematic search for infection, tissue injury, cytokine-driven inflammation, and disease activity rather than an inference that any one hematologic diagnosis is present (1, 3, 8, 11).

The acute leukemia analyses indicate that CRP may capture different components of systemic illness in AML and ALL. AML had a higher median CRP and lower hemoglobin and albumin, but the groups also differed substantially in age, and no adjusted model was available. Thus, the observed CRP difference cannot be attributed solely to lineage. More informative than the between-group comparison was the recurrent quartile pattern: increasing CRP was accompanied by decreasing albumin and increasing LDH in both AML and ALL. Albumin is influenced by inflammation, hepatic synthesis, capillary leak, and nutritional status, whereas LDH is a non-specific marker of cell turnover and tissue injury (3, 8, 10, 11). Their joint movement with CRP supports a composite systemic-burden phenotype, but not a direct measure of leukemia burden.

CML and CLL illustrate why CRP concentration and CRP context should be separated. Although median CRP did not differ, CML demonstrated broad associations with LDH, hemoglobin, and albumin, whereas the strongest reproducible CLL association was with albumin. These differences may reflect lineage, proliferative kinetics, disease phase, or unmeasured clinical composition. Similarly, HL showed higher CRP than the broad NHL category and displayed increasing leukocyte and platelet counts across CRP quartiles, compatible with a more pronounced acute-phase and reactive hematopoietic response in some patients. Because NHL encompasses biologically diverse entities (15) and because age and stage were not adjusted, these findings should be regarded as phenotype descriptions rather than subtype-discrimination rules.

The MM analysis provides an important negative finding. EMD is a clinically consequential manifestation of plasma-cell neoplasia, but CRP was identical in patients with and without EMD and the distributions overlapped extensively. Routine CRP therefore appears unsuitable as a stand-alone indicator of EMD. Imaging and, when appropriate, tissue confirmation remain necessary within the broader diagnostic assessment of MM (16). The absence of significant CRP–laboratory correlations in the smaller EMD subgroup may reflect biological heterogeneity, limited power, or differences in patient composition. Conversely, in non-EMD MM, the association of CRP with albumin, LDH, leukocyte count, and creatinine suggests that CRP can still summarize systemic inflammatory and organ-function disturbance without conveying anatomical information about extramedullary involvement.

The NHANES component provided an exploratory descriptive comparison but has major interpretive constraints. Participants were identified by self-reported physician diagnosis and could have active, remitted, or remote disease; histologic subtype, treatment, and time since diagnosis were unavailable. The non-cancer group was also considerably younger, and the analyses were unweighted and unadjusted. These factors prevent a claim of population representativeness or external validation of pretreatment hospital-cohort findings (17). The inverse correlations between hs-CRP and hemoglobin or albumin are directionally consistent with the clinical cohort, but similar associations occur in many inflammatory and chronic conditions.

Clinically, our results support a restrained, disease-aware interpretation of CRP. CRP may help clinicians quantify systemic inflammatory burden, identify discordance between an apparently indolent diagnosis and a highly inflammatory presentation, and contextualize anemia, hypoalbuminemia, or increased LDH. It should not be used in isolation to diagnose a hematologic disorder, define a universal risk threshold, identify EMD, or infer prognosis (1, 3, 8, 11). Accordingly, the disease-specific CRP quartiles used in this study should be interpreted as exploratory analytical strata rather than clinically validated CRP cutoffs. Combining serial CRP with disease-specific markers, stage, molecular risk, infection adjudication, imaging, and treatment response may be more informative than a single baseline value. Future work should evaluate whether dynamic CRP trajectories improve prediction beyond established disease-specific models.

This study has several strengths: a large cohort, broad disease coverage, pretreatment baseline sampling, clinically relevant subtype comparisons, and concordant correlation and quartile analyses. It also has important limitations. The single-center retrospective design introduces selection and documentation bias. Residual infection remains an important potential source of confounding. Although patients with documented acute bacterial or viral infection were excluded, occult or incompletely documented infection, including bacterial or fungal infection and febrile neutropenia, could not be reliably excluded, particularly in acute leukemia, HLH, and aggressive lymphoma; therefore, elevated CRP in these groups cannot be attributed solely to the underlying hematologic disorder. Disease-specific stage, cytogenetic abnormalities, molecular risk classification, and quantitative measures of disease burden were not consistently available across disorders, and these factors may influence systemic inflammation and CRP concentrations. The analyses were predominantly unadjusted and were designed as descriptive rather than causal; observed between-group differences may therefore also reflect differences in age, sex, disease severity, and other aspects of clinical composition. Body mass index, smoking, cardiovascular disease, treatment history, and other confounders were not consistently modeled. The long study period also raises the possibility of laboratory assay/platform and diagnostic-classification drift. Although the analytical principle for CRP remained immunoturbidimetric, complete historical records of individual analyzers and reagent manufacturers were not uniformly retrievable, so platform- or reagent-related variation cannot be fully excluded. Sex was unavailable for 528 patients, and other variables were analyzed using available cases. The analyses involved multiple comparisons. NHL and acute/chronic leukemia categories were heterogeneous, and no longitudinal CRP, response, relapse, or survival outcomes were available. Routine assays did not distinguish pentameric from monomeric CRP, so the data cannot establish CRP-mediated causality (2–7). Finally, the NHANES analysis was unweighted, unadjusted, based on self-reported history, and cannot function as a true external validation cohort.

## 5 Conclusion

Baseline CRP exhibits pronounced disease-specific heterogeneity across hematologic disorders. High concentrations are most evident in hyperinflammatory conditions and selected aggressive malignancies, whereas the laboratory phenotype accompanying CRP differs even between related subtypes. CRP does not distinguish MM with from MM without EMD. These findings support CRP as a context-dependent measure of systemic inflammatory burden, not as a stand-alone diagnostic, anatomical, or prognostic biomarker. Multicenter prospective studies incorporating standardized assays, longitudinal sampling, multivariable adjustment, disease-specific outcomes, and molecular characterization are needed before CRP can be incorporated into formal hematologic risk models.

## Data Availability

The raw data supporting the conclusions of this article will be made available by the authors, without undue reservation.
